# Health Risk Assessment of Total Suspended Particulate Exposure to Employee of PT Semen Padang, Indonesia

**Published:** 2019-08

**Authors:** Aria GUSTI, Resi Arifa YURNAL

**Affiliations:** Department of Environmental Health, Faculty of Public Health, Andalas University, Padang, Indonesia

## Dear Editor-in-Chief

The cement industry is now one of the sectors that play a role in regional and state economic development. The negative impact of this industrial activity is to cause air pollution inside or outside the work environment. One of the air pollutants that can cause health problems is rough or total suspended particulate (TSP) particles (1). According to WHO, a person exposed to TSP particulates may have acute respiratory infections (ARI), asthma, enfisema, lung cancer, cardiovascular disease, and chronic obstructive lung disease (2).

Perseroan Terbatas (PT) Semen Padang is known that the area is a high level of exposure to dust that can cause lung disease and respiratory symptoms (3). Based on data obtained health effects resulting from exposure to TSP has been experienced by many employees of the production department (3). TSP exposure plays a role in the increased risk of respiratory and lung diseases in workers (4). TSP in the Cement Industry contains many ingredients such as tricalcium silicate, dicalcium silicate, some alumina, tricalcium aluminate, iron oxide and a small amount of hexavalent chromium (5). In addition to the cement industry many silicone, ferro and lead particles. These different circumstances also provide different toxicological properties and levels that provide different health risks to the human body (6).

We aimed to determine the level of health risk due to exposure to TSP to employees of PT. Semen Padang, Indonesia. The results of this study are not only useful in risk control, but also can be used as a scientific framework in decision making and Policies to address health and environmental issues.

This research was a quantitative research in the form of descriptive method of environmental health risk analysis (EHRA) which aimed to calculate the level of risk received by a population due to the exposure of TSP in the environment. This study was conducted in 2016 with a total sample of 32 respondents. Respondents signed an informed consent and ethical approval was obtained from the faculty Ethics Committee.

Anthropometric data collection and activity pattern by interview using questionnaire and TSP concentration measurement using Staplex Model TFIA series High Volume Air Samplers (HVAS). Some of the procedures involved include hazard identification and risk sources, dose-response analysis, exposure assessment, and risk characterization. The risk level is expressed in the Risk Quotient (RQ) expressed as the ratio between the value of the intake and the reference dose (RfC). Intake is the amount of inhaled concentration per kilogram of body weight, while RfC is an approximate daily exposure dose that has no health effects in lifetime exposure. A situation is considered risky and management of control is required if RQ> 1.

The largest concentration of TSP was in the Cement Mill area of 40.8 mg / m3 and the smallest in the Raw Mill area of 11.9 mg / m3 (Table 1), the concentration value passed the inhalation particulate threshold value for the employees.

**Table 1:** Total suspended particulate concentration in air environment

***No.***	***Location***	***Distance***	***Time***	***Lenght of Measurement***	***consentration***
1	Raw mill	15 m	09.30–15.30	6 hour	11.9 mg/m^3^
2	Coal mill	15 m	08.43–15.43	7 hour	30.6 mg/m^3^
3	Kiln	15 m	09.00–16.00	7 hour	20.4 mg/m^3^
4	Cement mill	15 m	09.00–16.00	7 hour	40.8 mg/m^3^

Risk Quotient (RQ) for the TSP (inhalation) exposure of the lifetime intake value at four points there are two areas that are at risk of respiratory problems in employees in the Coal mill and Cement mill areas (Table 2). While RQ for TSP exposure (inhalation) of realtime intake values at four research sites did not risk respiratory distress in employees with an average weight of 66 kg, it has been exposed 270 days / year for 7 years and 4 months working in Production Department II / III PT Semen Padang.

**Table 2:** Values of Risk Quotient (RQ) for Lifetime and Realtime Intake

***Point of Sample***	***Lifetime***			***Realtime***		
	***Intake life time***	***RQ***	***Risk***	***Intake real time***	***RQ***	***Risk***
Raw mill	0.88	0.36	Not Risk	0.22	0.09	Not Risk
Coal mill	2.27	1.00	Risk	0.56	0.23	Not Risk
Kiln	1.52	0.62	Not Risk	0.37	0.15	Not Risk
Cement mill	3.03	1.25	Risk	0.70	0.30	Not Risk

The RQ value was calculated based on the duration of the lifetime and realtime exposure with RfC value of 2.42 mg/kg/d obtained from IRIS US-EPA data. Based on the description of health effects resulting from exposure to TSP has been experienced by many employees of production. TSP exposure plays a role in the increased risk of respiratory and lung disease diseases in workers. The risk control that can be done to reduce the particulate concentration of TSP in air emissions can be done with some controls, first by installing air filtering devices on pollutant sources in the factory area, secondly reducing the concentration can also be done by reducing the daily capacity of the production capacities.
